# Biomimetics and bioinspired surfaces: from nature to theory and applications

**DOI:** 10.3762/bjnano.16.32

**Published:** 2025-03-26

**Authors:** Rhainer Guillermo Ferreira, Thies H Büscher, Manuela Rebora, Poramate Manoonpong, Zhendong Dai, Stanislav N Gorb

**Affiliations:** 1 Lestes Lab, Federal University of Triângulo Mineiro, Uberaba, Minas Gerais, Brazil; 2 Department of Functional Morphology and Biomechanics, Kiel University, Am Botanischen Garten 1–9, 24098 Kiel, Germany; 3 Dipartimento di Chimica, Biologia e Biotecnologie, University of Perugia, Via Elce di Sotto 8, 06121 Perugia, Italy; 4 Embodied AI & Neurorobotics Lab, The Maersk Mc-Kinney Moller Institute, The University of Southern Denmark,Campusvej 55, 5230 Odense, Denmark; 5 State Key Laboratory of Mechanics and Control for Aerospace Structures, College of Mechanical and Electrical Engineering, Nanjing University of Aeronautics and Astronautics, Nanjing, China

**Keywords:** adhesives, bioengineering, biomimetics, drag reduction, functional morphology, insects, medical coatings, microstructures, nanostructures, wettability

The surfaces of living organisms are continuously interacting with their surroundings. As a result, they encounter a variety of challenges arising from both external and internal stimuli. Consequently, these surfaces must be multifunctional and adapt to numerous environmental pressures. Such pressures involve intricate interactions between surface structures and the environment across different scales, including nano-, micro-, and macroscales.

Biomimetics aims at making use of understanding how these adaptations and the particular material properties of these surfaces influence their performance and at drawing inspiration for modern technology from the vast array of solutions found in nature [1]. By examining the multiscale structures and mechanisms in biological systems, innovative and technologically advanced solutions can be developed for practical applications. Bioinspired nanotechnology plays a crucial role by harnessing nanoscale properties and processes to create highly effective surfaces and interfaces at various scales.

In May 2023, the Beilstein Nanotechnology Symposium “Functional Micro- and Nanostructured Surfaces: from Biology to Biomimetics” gathered diverse researchers from various disciplines in Limburg, Germany, to showcase important advances in biomimetics and discuss ideas providing an interdisciplinary platform to discuss novel developments and trends in the field of biological and bioinspired surfaces. This thematic issue in the *Beilstein Journal of Nanotechnology* emerged from this fruitful exchange of ideas.

The symposium featured a range of topics across biomimetic and bioinspired approaches, as well as the characterization of biological surfaces with properties of technological interest. A significant deal of research focused on understanding the biological systems and their potential as inspiration for innovation in producing biomimetic and bioinspired surfaces.

Key topics included bioinspired micro- and nanostructured surfaces, and their tribological properties like friction, wear resistance, and adhesion. Discussions also addressed surfaces with self-cleaning and wettability functionalities, as well as photonic surfaces, highlighting the broad sense and intent of the symposium for bridging biology and biomimetics in advanced materials sciences. The panel was composed of experts from all around the world resulting in the compilation of studies that form this thematic issue.

The thematic issue "Biomimetics and Bioinspired Surfaces: From Nature to Theory and Applications" is composed of nine articles that not only show the possibilities of analyzing natural phenomena in detail, but also empirical applications of bioinspired technology and new insights into the future of this field of research. Striking advance has been made regarding the study of surfaces on biological models, especially insects. For instance, insect attachment devices and adhesive secretions were thoroughly studied regarding the impact of contamination [2] and ageing [3]. Gorb and Gorb [2] experimentally investigated how different plant waxes affect the attachment performance of leaf beetles and how the adhesive system of these beetles are vulnerable to the shape and dimensions of wax contaminations. A study on larger insects (stick insects) with a considerable long-life expectancy by Grote et al. [3] focused on the structure and performance changes of the adhesive system during aging. The attachment performance of these insects decreases with increasing age, and was shown to be related to changes of the attachment pads regarding their elasticity, substrate compliance, and overall pad geometry. The attachment system of a second stick insect species was structurally investigated by Thomas et al. [4]. This article employed a range of imaging techniques to elucidate the ultrastructure and material composition of the two attachment pad types of this species.

Other possible sources of bioinspiration have been extensively examined by a review on functional surfaces in Hymenoptera, which include bees, wasps, and ants [5]. This diverse group of insects offers a rich array of surfaces that are adapted to realize different tasks, providing insights into the structure–function relationships of these surfaces useful for translational approaches. Further general insights into biological principles and their subsequent transfer into biomimetic engineering are provided in a multiscale biological analysis by Amador et al. [6], ranging from viruses to mammals while addressing the functional fibrillar interfaces in biological hair.

Presenting one applied example for biomimetic approaches, Ali et al. [7] used the hydrophobicity of the integument of spring tail (Collembola) as a template for the bioinspired development of nanofilament coatings that reduce scaling on steel surfaces. Using silicone nanofilaments, they achieved 75.5% reduction of calcium carbonate deposition on treated steel samples.

While many articles concentrated on using natural designs to inspire technological innovation (biology-push), others took an application-driven (technology-pull) approach. For instance, Bartoli et al. [8] reviewed the potential applications of nanostructured carbon coatings – such as nanodiamonds, carbon nanotubes, and graphene-based materials – to improve interaction on the interface between medical implants and living cells. Several biological materials exhibit microstructures that reduce drag; for instance, bees and wasps have structures on the wings that facilitate flying [5]. Zhu et al. [9] applied this concept in using microtextures to rotating blades of aircraft engines. Their results show that the microtextures may improve energy efficiency by 3.7% of a single blade by reducing the drag, which improves the overall performance of the engine. Finally, Sameoto [10] presents a stimulating perspective article on bioinspired adhesives, which advocates for a paradigm shift in biomimetics research. Instead of merely drawing inspiration from nature to discover new materials, the work proposes focusing on re-engineering applications to enhance manufacturing processes and improve the performance of biomimetic adhesives, thereby pushing the boundaries of this dynamic field further.

Overall, this thematic issue serves as an original resource of novel approaches and data regarding bioinspired surfaces, bridging biology and materials science. The high-quality contributions showcase innovative designs and practical applications of biomimetic surfaces. We express our gratitude to all the authors, who contributed their research to this collection, and to the reviewers, who helped us to critically discuss and improve the manuscripts. It is our hope that these studies inspire scientists, engineers, and innovators to further explore the possibilities of biomimetic designs, forging new paths in material science and technology.

## Dedication

**Figure 1:**
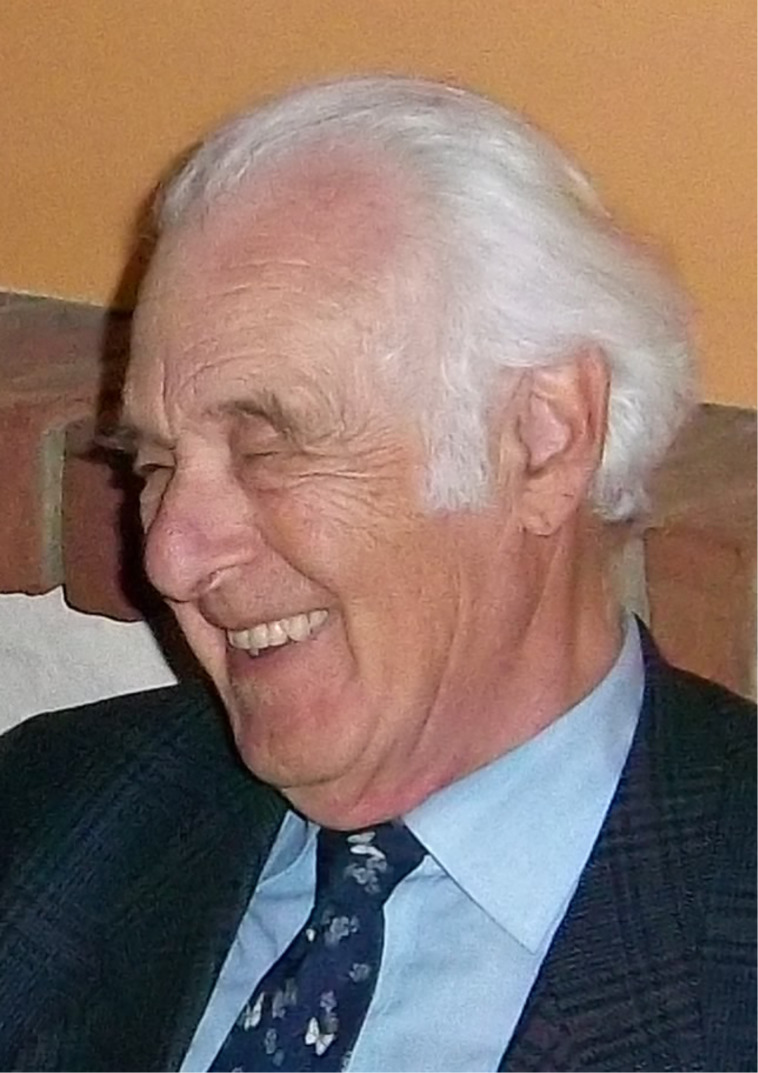
Werner Nachtigall during one of his visits in Kiel, 2012 (photograph by Stanislav N. Gorb). This content is not subject to CC BY 4.0.

Biomimetics, the key connective element of this thematic issue, was fundamentally influenced by Prof. Dr. Werner Nachtigall (Figure 1). He is considered one of the pioneers of this field and contributed with outstanding achievements in theory and praxis to biomimetics. Numerous books, studies on biological templates, and bioinspired applications have been published with his participation. Sadly, the biomimetic community lost this major personality on the 5th of September, 2024, who passed away at the age of 90 years old. We dedicate this thematic issue on biomimetic surfaces to commemorate his achievements and motivation.

Werner Nachtigall’s unparalleled commitment to establishing the concept of biomimetics and promoting bioinspired problem solving left clear marks. In 1990, he established the study curriculum *Technical Biology and Biomimetics* within the Biology program at Saarland University where he worked as full professor since 1969 and remained there until his retirement in 2002. He founded the *Society for Technical Biology and Biomimetics* of which he served as the first chairman until 2003 to support the field nationally and internationally, as well as the *Biomimetics Network of Excellence e.V.* (BIOKON).

Werner Nachtigall enriched the scientific community through various activities. He acted as a trusted lecturer for the German National Academic Foundation, as a long-time reviewer for the German Research Foundation, as a member of the Mainz Academy of Sciences and Literature, and as a member of the Sudeten German Academy of Sciences and Arts in Munich. We received the news of the passing of Werner Nachtigall with great sadness during the compilation of this thematic issue. With more than 300 publications, he remains visible in the field and the memories of biomimeticists, as highlighted in detail in an obituary by Reihnard Blickhahn [11]. Most noteworthy, he will be remembered for his comprehensive books on the conceptualization and establishment of biomimetics as a scientific discipline, such as *Biomechanik* (biomechanics) [12], *Bionik – Grundlagen und Beispiele für Ingenieure und Naturwissenschaftler* (Biomimetics – basics and examples for engineers and scientists) [13] or *Bionik als Wissenschaft* (Biomimetics as a scientific discipline) [14] which remain influential for the scientific community. His book on biological attachment mechanisms and their use in bioengineering has been influential for scientists working on biomimetic surfaces since the 1970 [15] and paved the way for one of the core topics of this thematic issue.

Rhainer Guillermo Ferreira, Thies H. Büscher, Manuela Rebora, Poramate Manoonpong, Zhendong Dai and Stanislav N. Gorb

Uberaba, Kiel, Perugia, Odense, and Nanjing, January 2025

## Data Availability

Data sharing is not applicable as no new data was generated or analyzed in this study.
